# Investigating the Influence of Diffusion on the Cohesive Zone Model of the SiC/Al Composite Interface

**DOI:** 10.3390/molecules28196757

**Published:** 2023-09-22

**Authors:** Masoud Tahani, Eligiusz Postek, Tomasz Sadowski

**Affiliations:** 1 Department of Mechanical Engineering, Ferdowsi University of Mashhad, Mashhad 9177948974, Iran; 2 Institute of Fundamental Technological Research, Polish Academy of Sciences, Pawińskiego 5B, 02-106 Warsaw, Poland; epostek@ippt.pan.pl; 3 Department of Solid Mechanics, Lublin University of Technology, 20-618 Lublin, Poland; t.sadowski@pollub.pl

**Keywords:** metal matrix composite, diffusion, cohesive zone law, interface, molecular dynamics

## Abstract

Modeling metal matrix composites in finite element software requires incorporating a cohesive zone model (CZM) to represent the interface between the constituent materials. The CZM determines the behavior of traction–separation (T–S) in this region. Specifically, when a diffusion zone is formed due to heat treatment, it becomes challenging to determine experimentally the equivalent mechanical properties of the interface. Additionally, understanding the influence of heat treatment and the creation of a diffusion zone on the T–S law is crucial. In this study, the molecular dynamics approach was employed to investigate the effect of the diffusion region formation, resulting from heat treatment, on the T–S law at the interface of a SiC/Al composite in tensile, shear, and mixed-mode loadings. It was found that the formation of a diffusion layer led to an increase in tensile and shear strengths and work of separation compared with the interfaces without heat treatment. However, the elastic and shear moduli were not significantly affected by the creation of the diffusion layer. Moreover, the numerical findings indicated that the shear strength in the diffusion region was higher when compared with the shear strength of the {111} slip plane within the fcc aluminum component of the composite material. Therefore, in the diffusion region, crack propagation did not occur in the pure shear loading case; however, shear sliding was observed at the aluminum atomic layers.

## 1. Introduction

Metal matrix composites (MMCs) are advanced materials that combine the benefits of metals and reinforcements with superior mechanical properties compared with traditional monolithic metals. The MMCs consist of a metal matrix reinforced with ceramic particles, fibers, or other reinforcement shapes. The MMCs offer exceptional mechanical and physical properties, making them attractive for various aerospace, automotive, and biomedical engineering applications.

The performance of MMCs largely depends on the mechanical properties of the constituents and the equivalent mechanical characteristics of the interface between the reinforcing phase and the metal matrix [1,2]. The interface is a load transfer zone, where stress is transferred between the matrix and the reinforcement. A weak or poorly bonded interface can lead to premature failure and compromised mechanical performance of the composite. On the other hand, an adequately bonded interface can significantly enhance the mechanical properties of the composite, leading to improved strength, stiffness, toughness, and fatigue resistance. Therefore, characterizing the interface’s equivalent mechanical properties has become a research interest and is essential for the effective design and development of high-performance MMCs. The importance of the interface characteristics among the phases in polycrystalline composites was demonstrated in previous studies (for example, see Refs. [3,4]).

The complexity of the interface arises from the inherent distinctions between the properties of the matrix and the reinforcing phase. The metallic matrix typically has high ductility and good formability, while the ceramic reinforcement frequently has high strength, stiffness, and thermal stability. Achieving a strong and stable interface that can effectively transfer loads between these two dissimilar phases is challenging. Various technological methods have been proposed to enhance the wetting of ceramics by liquid metal, given that ceramics typically have poor wettability with metals. These approaches include elevating the temperature of the liquid metal, pre-treating ceramic particles or fibers, applying coatings to the ceramics, and introducing surface-active elements into the matrix. The primary emphasis of this study was investigating the influence of heating the composites to create a diffusion layer. The system underwent a cooling process following the heating phase to assess the overall mechanical properties of the interface using the T–S law in this region.

The cohesive zone law defines the T–S relationship along the interface. It characterizes the stress required to initiate and propagate cracks or delamination in the interface. The T–S law is derived from experimental or numerical data and provides a fundamental relationship that quantifies the mechanical response of the interface to external loading. It defines the cohesive properties of the materials and their resistance to failure. Much research has been published in the literature investigating the CZM for the interface zone of materials. For instance, Zhou et al. [5] considered the bilayer structure of brittle materials and studied the interfacial crack growth at the interface under mixed-mode loadings using MD. Noreyan et al. [6] determined the critical shear stress and plasticity flow at Si/Al and Al/Al interfaces. They found that the critical shear stress was significantly lower than critical tensile stress at the Si/Al interface, and the fracture was mainly localized within the Al close to the interface. Dandekar and Shin [7] obtained the T–S law for the SiC/Al interface under tensile and shear loadings at different temperatures using MD simulations. Yang et al. [8] analyzed crack propagation at the SiC/Cu interface under mixed-mode loading utilizing the MD method. They compared the crack propagation behavior for the weak and strong interfaces with different interface energy by adjusting the interaction parameters between SiC and Cu. Zhou et al. [9] analyzed the crack propagation at the SiC/Mg interface under mixed-mode loading using MD simulations. Their findings revealed the presence of four distinct asymmetrical crack propagation modes in the SiC/Mg interface. They also observed that strong interface bonding conditions led to both brittle and ductile crack propagation modes; however, weak interfaces exhibited solely brittle failure. Fathalian et al. [10] recently investigated the interfacial behavior and fracture mechanism of the C- and Si-terminated 6H-SiC/Al interfaces with vacancy defects using the density functional theory calculations. They determined the work of adhesion and the fracture toughness of the interfaces and concluded that the Si-terminated interfaces had a lower fracture toughness than the C-terminated ones.

As previously mentioned, one technique to improve the wettability of ceramics with metals involves raising the temperature and liquefying the metal. To the best of our knowledge, no study has yet explored the influence of temperature elevation on subsequently determining the equivalent mechanical properties of the interface. Increasing the temperature will form a thin diffused region, which will significantly impact the nature and strength of the interface. Many researchers have investigated the interdiffusion phenomenon for different diffusion couples through heating systems. For brevity, only a selection of studies on this subject will be mentioned here. Chang et al. [11] examined the diffusion phenomenon in the field of semiconductors and measured the diffusion coefficient within the temperature range of 1700 to 2400 °C. Tajima et al. [12] calculated the diffusion constant of Al into SiC at a temperature range of 1350 to 1800 °C. Sozhamannan and Prabu [13] fabricated samples with SiC/Al interface bonding at different processing temperatures and constant holding times. The interface compounds were analyzed using an energy-dispersive spectroscope. The diffusion length of the compounds was then computed, and the interface characteristics were assessed through tensile and microhardness tests.

Müting et al. [14] examined the diffusion of Al in 6H-SiC during postimplantation annealing. Using defect-enhanced diffusion mechanisms, they discovered that during the heat treatment, Al diffuses in SiC at a low rate. Tahani and coworkers [15,16] investigated the diffusion and interdiffusion at the C- and Si-terminated SiC/Al interfaces. They examined pristine SiC and SiC with vacancy defects to study the interdiffusion. They determined the thickness of the diffusion zone as a function of annealing temperature and time and the main and interdiffusion coefficient for these ternary systems. Luo et al. [17] simulated the interdiffusion between Mo and Ti, revealing asymmetry in the process. Mo atoms diffused more readily into the Ti region than Ti atoms into the Mo block. Ouyang et al. [18] investigated the effect of temperature on the diffusion bonding process of the Ni–Zr interface. They found that the thickness of the diffusion zone increased with the temperature and time. Furthermore, Zhu et al. [19] used molecular dynamics (MD) simulations to study the interdiffusion between metallic glass CuZr and crystal Al. They observed significant asymmetry in the interdiffusivity between the neighboring amorphous and crystalline phases, with a noticeable concentration dependence. Moreover, Tahani et al. [20] investigated the interdiffusion at the Al- and O-terminated Al_2_O_3_/AlSi12 interfaces. They determined the diffusion constants and interdiffusion coefficients for the ternary system.

This paper examines the crack propagation behavior at the SiC/Al interface under modes I, II, and mixed-mode loading conditions. First, the SiC/Al composite was heated at 1500 K for 6.0 ns to create a diffusion layer. Subsequently, the system was cooled to 300 K before undergoing mechanical loading to establish T–S relationships. The effect of the heat treatment on the cohesive zone parameters of the interface was examined by comparing the interfaces without the heat treatment with those subjected to the heat treatment. Additionally, the study explored the influence of the type of atom termination at the interface and the cubic or hexagonal crystal structure of SiC on the T–S behavior.

## 2. Simulation Results and Discussion

In order to examine the effect of heat treatment on the equivalent mechanical properties of the interface, two types of interface (i.e., before and after heat treatment for diffusion) were studied. Furthermore, the influence of the atom termination at the interface and the cubic or hexagonal crystal structure of SiC was investigated on the interface characteristics. The composite interfaces were subjected to modes I, II, and mixed-mode loading conditions.

### 2.1. Crack Growth Process

**Figure 1:**
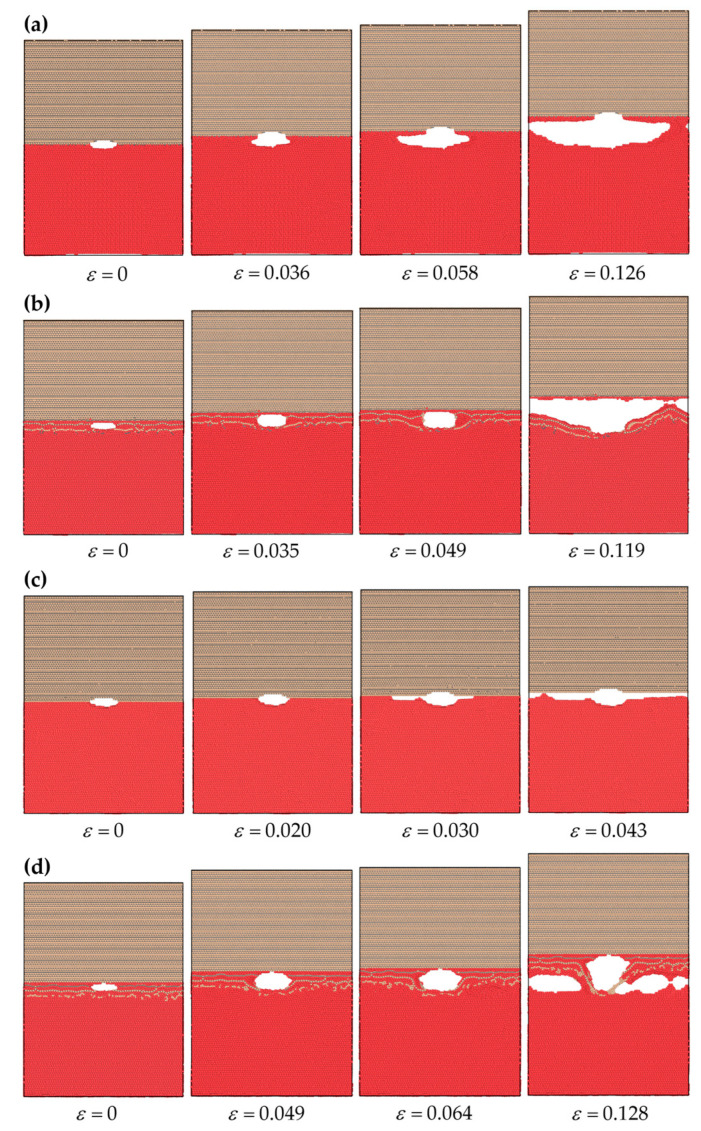
Snapshots of crack propagation at interface models under pure tensile loading (mode I). The C-terminated 6H-SiC/Al (**a**) before and (**b**) after DL formation. The Si-terminated 6H-SiC/Al (**c**) before and (**d**) after DL formation.

Atomic snapshots of the C-terminated 6H-SiC/Al interface before and after diffusion layer (DL) formation under pure tensile loading (mode I) are shown in Figure 1a,b, respectively. Similar results are also illustrated in Figure 1c,d for the Si-terminated 6H-SiC/Al interface before and after heat treatment. Each figure begins with the model at the initial state (*ε* = 0), followed by the model when the normal stress was at its maximum, the model in a plastic region before rupture, and finally, the model at the rupture point. The Young modulus of SiC is about 314.2 MPa [21], whereas Al has a Young modulus of 63.8 GPa [22]. It implies that in the SiC/Al composite system, the SiC part of the models behaves as a nearly rigid body compared with the Al part. This observation aligns with the findings depicted in Figure 1, where the majority of deformation was observed in the Al component of the model. The lattice mismatch affected several layers of atoms around the interface, both in Al and SiC. The SiC–Al interaction constrained the layers of Al atoms near the interface, resulting in distinct characteristics compared with the bulk Al atoms. Therefore, the Al atoms in the interfacial region exhibited notable differences compared with their counterparts in the bulk material. 

It is observed from Figure 1a,c that the Si-terminated interface was weak compared with the C-terminated one. As a result, the crack propagated primarily along the interface for the Si-terminated case. In contrast, for the C-terminated interface, the crack propagated within a few layers of the Al rather than along the interface. This observation was consistent with the findings of Hoekstra and Kohyama [23]. They showed that the bonds between C and Al exhibited a higher degree of localization in their charge distribution at the interface, and consequently, these bonds were nearly twice as strong as the more dispersed Si and Al bonds. Moreover, the present results were in line with the observations made by Wang et al. [24], which highlighted the strong interfacial bonds and adhesive capacity of the C-terminated interface as opposed to the Si-terminated counterpart.

Figure 1b,d illustrate that a diffusion region at the interface was generated after heat treatment, and a fuzzy interface was produced instead of a sharp one. Based on the observations from these figures, it can be inferred that the absence of a distinct interface, particularly in the Si-terminated case, due to diffusion delayed the separation at the interface. As a result, the sample exhibited an increased ability to withstand a more significant amount of tensile strain before experiencing rupture.

The numerical results indicated that in the C-terminated interfaces before DL formation and all cases after DL formation, the failure mechanism followed a progression characterized by crack tip blunting, initial crack growth, void nucleation, and void growth and coalescence. However, in Si-terminated interfaces, before DL formation, crack propagation and subsequent separation occurred at the interface due to weak interface bonding.

For brevity, similar atomic snapshots for the Si- and C-terminated 3C-SiC/Al interface are not included here, but the traction–separation law for all cases is provided.

### 2.2. Traction–Separation Response

**Figure 2:**
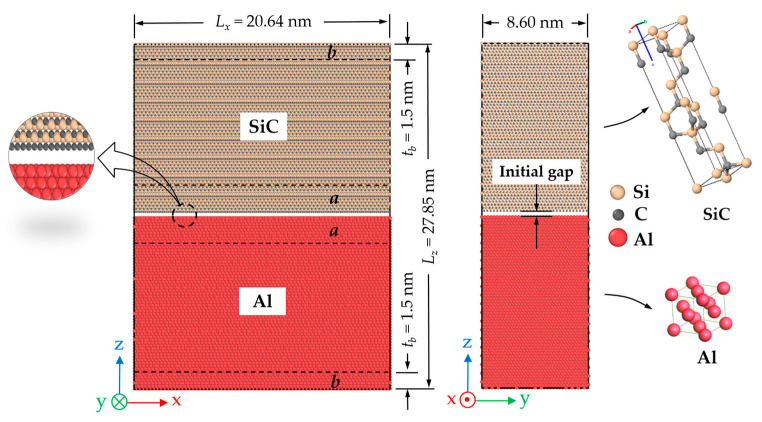
Schematic view of the C-terminated 6H-SiC/Al simulation model, dimensions, and coordinated system.

Zhou et al. [5] suggested using a narrow region near the interface (crack) represents the local traction and displacement measurements. Therefore, to obtain the traction–separation relation for the CZM, the normal and shear stress components *σ_z_* and *σ_xz_* were obtained by averaging the atomic virial stress at a distance of ±30 Å near the crack (i.e., region *a* in Figure 2). The separation or crack opening displacement was also determined by calculating the average displacement of atoms within region *a* in Figure 2. The opening displacements in the normal direction (Δ*z*) and the shear direction (Δ*x*) were defined and measured as the average displacements of atoms in the upper half of the region compared with those in the lower half. The magnitude of the total crack opening displacement was defined as $\Delta r=\sqrt{\Delta x^{2}+\Delta z^{2}}$.

**Figure 3:**
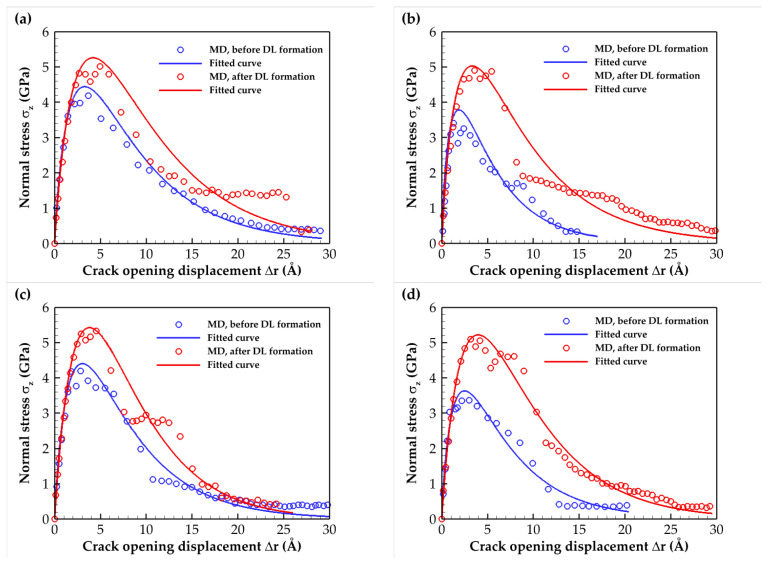
Traction–separation relation for the SiC/Al composite under tensile loading for both cases before and after DL formation. The (**a**) C- and (**b**) Si-terminated 6H-SiC/Al interfaces. The (**c**) C- and (**d**) Si-terminated 3C-SiC/Al interfaces.

The normal stress T–S results for the C- and Si-terminated 6H-SiC/Al and 3C-SiC/Al subjected to tensile loading (mode I) for interfaces before and after DL formation are illustrated in Figure 3. The constitutive relation of the SiC/Al interface is also described using an exponential cohesive zone law [25]. Therefore, the fitted curve, in addition to the MD results, is presented in Figure 3. The selected fitted function is as follows:

(1) $$\sigma =\sigma_{\max}\frac{\Delta r}{\delta_{0}}\exp\left\{\left[1-{\left(\frac{\Delta r}{\delta_{0}}\right)}^{\alpha }\right]/\beta \right\}$$

where *σ*_max_ indicates the maximum normal stress, *δ*_0_ is the corresponding displacement, and the additional parameters *α* and *β* control the overall shape and the decay rate of the stress.

The simulation results for the elastic modulus, tensile strength, toughness, and work of separation, comparing models without and with heat treatment for the mode I fracture test, are presented in Table 1. For all models, the elastic modulus remained nearly constant, indicating minimal influence from diffusion. The slight change in the modulus of elasticity can be attributed to the fact that the diffusion layer was very thin compared with the thickness of the SiC and Al. The obtained elastic modulus for the cracked models was very close to the experimental value of about 167 ± 10 GPa [26,27]. Furthermore, in both SiC polytypes, the heat treatment led to notable enhancements in the tensile strength, toughness, and work of separation at the interface. Specifically, compared with the untreated interface, the tensile strengths were increased by approximately 20% and 40% for the C- and Si-terminated surfaces, respectively. Additionally, the work of separation experienced significant improvements, increasing by approximately 30% and 100% for the C- and Si-terminated interfaces, respectively. These enhancements highlighted the positive effect of the heat treatment on the interface’s equivalent mechanical properties during mode I fracture.

In order to estimate the overall validity of the findings, the following analytical Griffith’s theory of fracture for the failure of brittle materials can be used [28]:

(2) $$\sigma_{\max}=\sqrt{\frac{E\;\cdot \;woa}{\pi \;\cdot \;a_{h}}}$$

where *σ*_max_ is the critical stress of fracture, *E* is the elastic modulus of the cracked specimen, *a_h_* is the crack half length, and *woa* is the work of adhesion. The work of adhesion is defined as [5]

(3) $$woa=\Gamma_{SiC}+\Gamma_{Al}-\Gamma_{SiC/Al}$$

where Γ*_SiC_* and Γ*_Al_* are the surface energies of the SiC and Al, respectively, and Γ*_SiC/Al_* is the interfacial energy between SiC and Al. The surface and interfacial energies are defined as follows:

(4) $$\Gamma_{i}=\frac{1}{2A_{1}}(E_{i}^{surf}-E_{i}^{bulk}),\;\Gamma_{SiC/Al}=\frac{1}{2A_{2}}[E_{SiC/Al}-(E_{SiC}+E_{Al})]$$

where *i* is SiC or Al, $E_{i}^{surf}$ and $E_{i}^{bulk}$ are the energy of the system *i* with and without the surface, respectively, and *A*_1_ and *A*_2_ are the surface area for computing the surface energy and the area of the interface, respectively (e.g., see [29]). Furthermore, *E_SiC/Al_*, *E_SiC_*, and *E_Al_* are the total energies of the SiC/Al interface, bulk SiC, and bulk Al after relaxation, respectively. It is worth noting that the surface and interfacial energies were computed for the 6H-SiC/Al and 3C-SiC/Al interfaces with the orientation relationships $\left.(0001)[2\bar{1}\bar{1}0{]}_{\alpha -\mathrm{SiC}}\right\|\;\left(111\right)$[110]_Al_ and $\left.{\left(111)[01\bar{1}\right]}_{\beta -\mathrm{SiC}}\;\right\|\;{\left(111)[01\bar{1}\right]}_{\mathrm{Al}}$, respectively (see Section 3.2).

The work of adhesion values for the C- and Si-terminated 6H-SiC/Al and 3C-SiC/Al interfaces at 300 K (before DL formation) were obtained and are presented in Table 2. It was observed that these values were within the range of 0.743 to 1.3 J/m^2^ as evaluated by previous researchers [30,31]. It can also be observed that the work of adhesion was smaller than the work of separation in Mode I, as presented in Table 1, which was an expected result. Based on Young’s modulus values obtained from Table 1 and an initial crack length of 40 Å, Equation (2) predicts the fracture stresses presented in Table 2. The data presented in Table 1, obtained from MD simulations, showed reasonable consistency with the results obtained using Griffith’s theory in Table 2. However, the critical fracture stresses derived from atomistic simulations were slightly lower than those predicted by the analytical Griffith’s theory.

**Figure 4:**
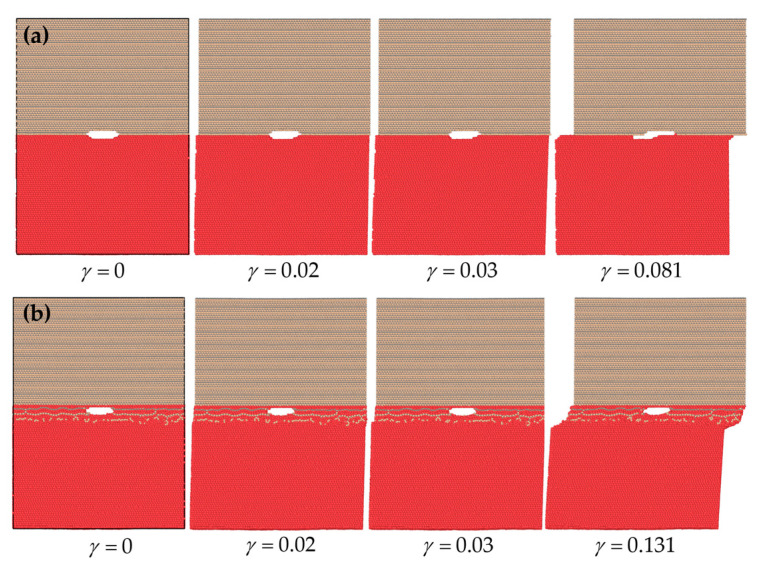
Snapshots of the Si-terminated 6H-SiC/Al interface models under shear loading (mode II). (**a**) Before and (**b**) after DL formation.

Next, the models were analyzed under mode II of fracture. Figure 4, for example, illustrates snapshots of the Si-terminated 6H-SiC/Al interface before and after DL formation. It can be seen that diffusion transformed the initially weak Si interface into a stronger one for shear loading conditions. As a result, the interface became more capable of withstanding higher shear strain levels.

In the models, especially after diffusion, the crack did not propagate under the shear loading mode because the crystallographic plane most commonly associated with shear slip in fcc aluminum was the plane. The family planes with face diagonal directions in the <110> family were close-packed in the fcc crystal structure and had a high density of close-packed planes and atoms. This arrangement allowed for easy dislocation motion and shear deformation to occur. The present crystallographic orientation relationship of aluminum in contact with cubic and hexagonal silicon carbide was a (111) plane. Hence, slipping in fcc Al happened. The Al crystal was not fcc in the interface region, especially after diffusion. However, the Al crystal structure was fcc within approximately 10 Å near the interface, and shear slip happened at those layers. Hence, the results for the traction–separation response are not presented for the pure shear loading case.

Table 3 shows the shear modulus and maximum shear stress for cases before and after DL formation. It was observed that the change of the shear modulus after diffusion was low. Furthermore, the maximum shear stress increased for the Si-terminated interfaces, where we had a weak interface before DL formation. For all interfaces, the maximum shear stress presented in Table 3 was significantly lower than the corresponding maximum tensile stress presented in Table 1 due to the occurrence of stick–slip during sliding. Noreyan et al. [6] made a similar observation in their research concerning the Al/Si interfaces. Notably, they found that the Si(111)/Al(111) interface exhibited the highest tensile strength while displaying the lowest shear strength among these interfaces. They concluded that in contrast to isotropic materials, where the critical shear stress was approximately half of the critical tensile stress, there was no direct relationship between shear and tensile critical stresses for nanostructures with different crystallographic orientations.

Next, it is intended here to determine the T–S behavior and the cohesive zone parameters of the SiC/Al interface under mixed-mode loading conditions. The simulations were conducted under seven distinct loading angles *θ*. The loading angles 0° and 90° represented pure tensile loading and pure shear loading, respectively. Any other loading angles indicated inclined tension loading, combining tensile and shear loading components. The specific loading angles and the corresponding rates of the boundary displacement are summarized in Table 4.

**Figure 5:**
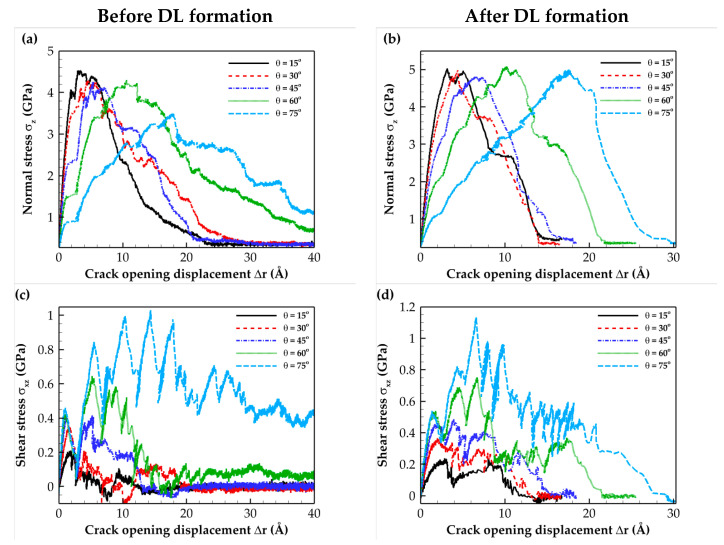
Traction–separation curves for the C-terminated 6H-SiC/Al interface under different mixed-mode loadings. Normal stress (**a**) before and (**b**) after DL formation. Shear stress (**c**) before and (**d**) after DL formation.

**Figure 6:**
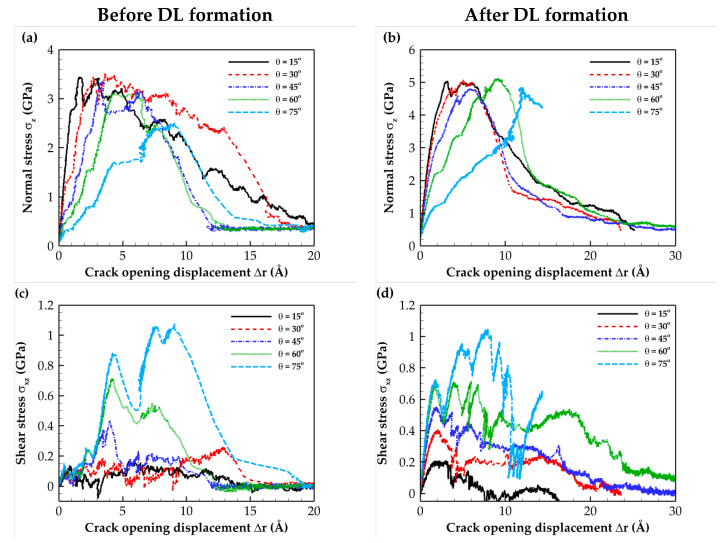
Traction–separation curves for the Si-terminated 6H-SiC/Al interface under different mixed-mode loadings. Normal stress (**a**) before and (**b**) after DL formation. Shear stress (**c**) before and (**d**) after DL formation.

**Figure 7:**
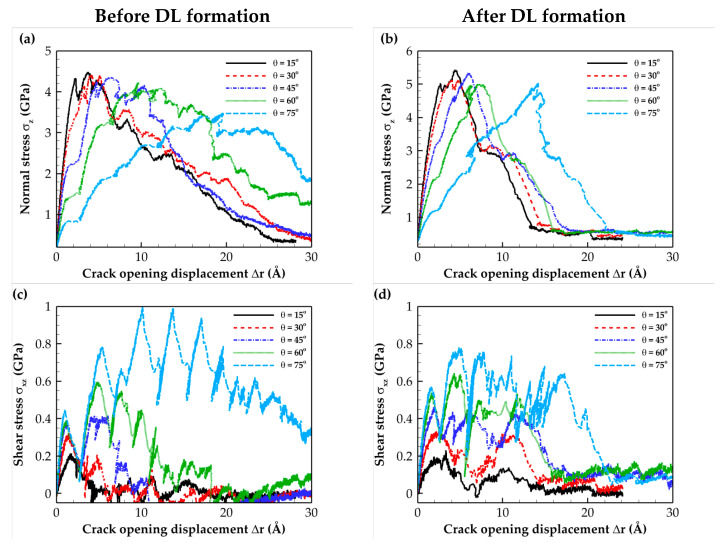
Traction–separation curves for the C-terminated 3C-SiC/Al interface under different mixed-mode loadings. Normal stress (**a**) before and (**b**) after DL formation. Shear stress (**c**) before and (**d**) after DL formation.

**Figure 8:**
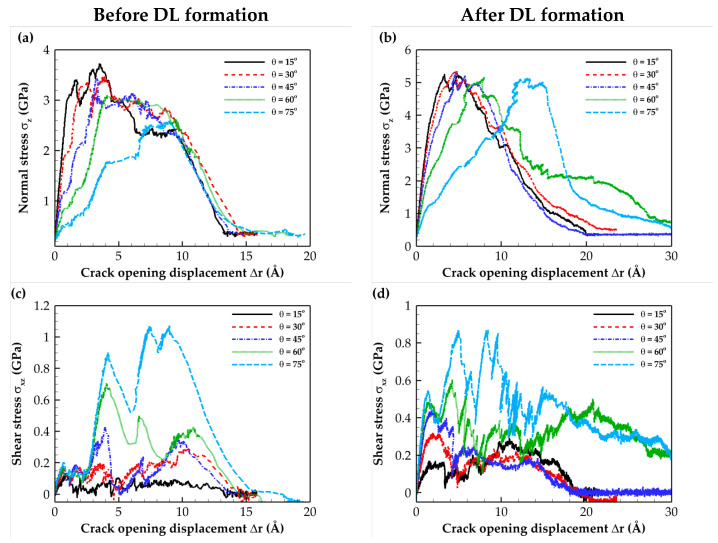
Traction–separation curves for the Si-terminated 3C-SiC/Al interface under different mixed-mode loadings. Normal stress (**a**) before and (**b**) after DL formation. Shear stress (**c**) before and (**d**) after DL formation.

The tensile and shear T–S curves for mixed-mode loadings with different loading angles for the C- and Si-terminated 6H-SiC/Al and 3C-SiC/Al composite interfaces are illustrated in Figure 5, Figure 6, Figure 7 and Figure 8. These plots indicate that as expected, the simulated system experienced a combination of tensile and shear stress during mixed-mode loading conditions. As the loading angle increased, the shear component became more dominant, leading to a rise in shear stress strength while the tensile stress strength decreased. In other words, an increase in the loading angle resulted in a stronger resistance to shear stress but a reduced resistance to tensile stress.

The normal stress T–S curves before and after DL formation for all interface types revealed that subjecting the composite to heat treatment increased the maximum normal stress. This change can be attributed to the formation of a diffusion region after heat treatment.

According to the data for the shear T–S curves, heat treatment does not significantly affect the maximum shear stress. This observation can be attributed to the shear slip in (111) Al planes, which is the shear failure mechanism. The sudden shear stress drop observed on the T–S curves resulted from shear slip occurring at the aluminum (111) planes, which is the primary deformation mechanism. Furthermore, when comparing the numerical separation values, it was evident that the Si-terminated interface tolerated lower separation than the C-terminated interface. This difference can be attributed to the weaker strength of Si-terminated interface than the C-terminated interface. The obtained results were consistent with the fact that the Al–Si bond strength is weaker than the stronger Al–C bond [23]. Finally, it is essential to highlight that the results presented in Figure 6b,d, which pertain to the Si-terminated 6H-SiC/Al interface under mixed-mode loading at *θ =* 75°, demonstrate that full crack propagation leading to rupture was not observed due to the sliding of Al atoms within the (111) planes.

### 2.3. Cohesive Zone Model

The cohesive zone model is a computational framework that simulates materials’ crack growth and fracture propagation. The CZM simplifies the fracture process zone as a region with a negligible thickness comprising two coincide cohesive surfaces. The following exponential law is proposed based on the T–S relationships presented previously:

(5) $$\sigma =\sigma_{\max}\lambda \exp\left(1-\lambda^{\alpha }/\beta \right)$$

where

(6) $$\lambda =\sqrt{{\left(u_{n}/\delta_{n}\right)}^{2}+{\left(u_{t}/\delta_{t}\right)}^{2}}$$

In Equation (5), *σ*_max_ is the maximum cohesive strength, *λ* is a non-dimensional parameter defined in Equation (6), and *α* and *β* are constants that will be determined using the MD results. Also, in Equation (6), *u_n_* and *u_t_* are the normal and tangential separations, and *δ_n_* and *δ_t_* are the maximum allowable normal and tangential separations, respectively. The cohesive element reaches failure when the value of *λ* equals 1.

**Figure 9:**
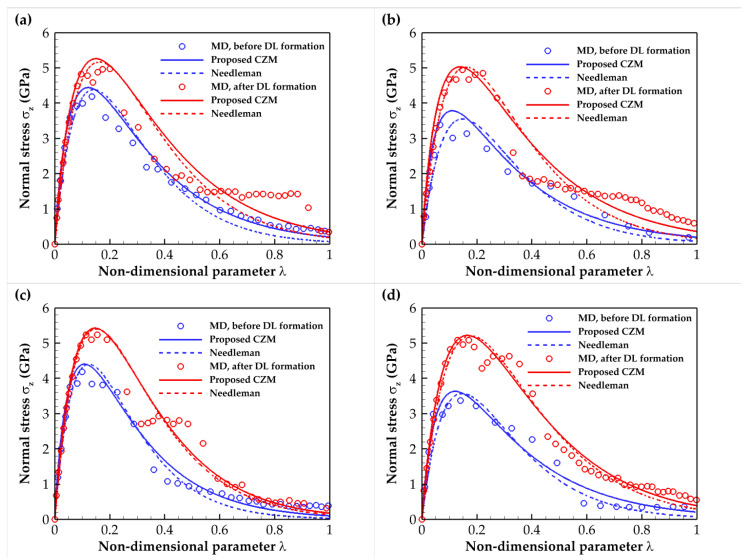
Comparison of the proposed CZM with the present MD results and continuum-based CZM models formulated by Needleman [32]. The (**a**) C- and (**b**) Si-terminated 6H-SiC/Al interfaces. The (**c**) C- and (**d**) Si-terminated 3C-SiC/Al interfaces.

Figure 9 compares the proposed CZM model of mode I failure with the present MD result and that obtained by the continuum-based CZM model of Needleman [32]. The hexagonal and cubic SiC/Al interfaces with the C- and Si-terminations for the cases before and after DL formation were examined. Table 5 lists the maximum stress, maximum separation, and constants *α* and *β* of the proposed CZM. These constants were obtained by minimizing the sum square difference between the MD results and the proposed CZM. It can be seen that the present CZM models aligned closely and exhibited notable consistency with those formulated by Needleman [32].

Furthermore, in all SiC/Al interfaces, the slope of the proposed model was marginally higher than the slope of the existing model in the elastic region. Additionally, the suggested model forecasted a slower interface degradation than the existing model. Once more, it was observed that diffusion enhanced the cohesive strength of the interfaces, with a more pronounced effect in the Si-terminated configuration in contrast to the C-terminated counterpart.

## 3. Simulation Methodology

As stated previously, the primary objective of this study was to develop a cohesive zone model for predicting the equivalent mechanical properties of the SiC/Al interface following heating and the formation of a diffusion region. Due to the extremely thin nature of the diffusion region, employing conventional continuum mechanics to develop a CZM becomes impractical. Hence, conducting atomistic simulations at the nanoscale is essential to capture the overall material behavior under specified conditions. The CZM gained from this modeling can then be utilized to construct a continuum model for the macroscale, employing a multiscale approach.

In this study, molecular dynamics simulations were conducted using the open-source program large-scale atomic/molecular massively parallel simulator (LAMMPS) package [33], and in order to visualize the evolution of the atomic structure, the open visualization tool (OVITO) [34] was utilized. In the molecular dynamics method, atomic interactions are needed to obtain the kinematic parameters corresponding to the particles using Newton’s second law. Therefore, the atomistic model should incorporate appropriate potential functions representing atomic interactions. The parameters of a potential function can be derived from ab initio calculations or experimental data such as the elastic modulus and cohesive energy. In the subsequent section, the interatomic potentials pertaining to aluminum, silicon carbide, and the interface will be explained.

### 3.1. Potential Functions

The embedded atom method (EAM) potential is widely used in simulations of metallic systems, allowing for the accurate exploration of various mechanical, thermal, and chemical properties. Incorporating this potential into the atomistic model makes it possible to represent the cohesive forces, elastic properties, and other atomic-scale phenomena specific to metallic materials accurately. In the present study, the EAM potentials proposed by Mendelev et al. [35] and Mishin et al. [36] were used to model the interaction between aluminum atoms. The interested reader will find definitions of the total energy in a monoatomic system in these EAM potentials in Refs. [35,36]. 

The Tersoff potential [37] is widely used in the literature for interactions between Si and C atoms. This potential is a bond-order potential, where the potential energy between atoms *i* and *j* is expressed as

(7) $$E=\sum_{i}E_{i}=\frac{1}{2}\sum_{i\neq j}f_{C}(r_{ij})\left[f_{R}(r_{ij})+b_{ij}f_{A}(r_{ij})\right]$$

where *r_ij_* represents the bond length between atoms *i* and *j*, and *f_R_*(*r_ij_*), *f_C_*(*r_ij_*), and *f_A_*(*r_ij_*) are the repulsive and attractive atomic pair interactions and the optional cutoff function that determines the interaction range, respectively. Also, *b_ij_* is a function that modulates the attractive interaction and incorporates many-body interactions.

The Morse potential was used for the SiC/Al interface, which is a two-body pair-wise potential. The Morse potential is expressed as

(8) $$V=D_{0}\left[e^{-2\alpha (r-r_{0})}-2e^{-\alpha (r-r_{0})}\right]$$

where *r*, *r*_0_, *D*_0_, and *α* are the distance between atoms, the equilibrium bond length, the well depth of the potential, and the width of the potential, respectively. The parameters of the Morse potential for the Al–C and Al–Si interactions are provided in Refs. [15,16,38].

The elastic constants of fcc aluminum and cubic silicon carbide (3C-SiC) were determined using the previously mentioned potential functions. The lattice parameters for fcc Al and 3C-SiC were 4.0495 Å and 4.348 Å, respectively. These elastic constants were then compared with experimental data and molecular dynamics simulations, as shown in Table 6. Young’s modulus, Poisson’s ratio, shear modulus, and bulk modulus were calculated using the elastic constants (e.g., see Refs. [15,16]). The results demonstrated excellent agreement between our findings and those obtained from experiments and MD simulations conducted by other researchers. This agreement indicates that the potential functions utilized in this study effectively captured the interactions between atoms, validating their suitability for modeling such interactions.

### 3.2. Molecular Dynamics Model

In this study, both cubic (3C-SiC) and hexagonal (6H-SiC) forms of SiC with a higher likelihood of orientation relationships were modeled as separate samples, each representing a different SiC crystal polytype. The α-SiC has a hexagonal crystal structure, while the β-SiC has a cubic crystal structure. For the Al composites reinforced with SiC particles, the orientation relationship $\left.(0001)[2\bar{1}\bar{1}0{]}_{\alpha -\mathrm{SiC}}\right\|\;\left(111\right)$[110]_Al_ was considered for the α-SiC particulate-reinforced Al [41]. On the other hand, the orientation relationship $\left.{\left(111)[01\bar{1}\;\right]}_{\beta -\mathrm{SiC}}\;\right\|\;{\left(111)[01\bar{1}\right]}_{\mathrm{Al}}$ was considered for the β-SiC whisker-reinforced Al [42].

It is worth mentioning that the generation of the grain boundaries involved rotating the two crystals around the appropriate rotation axis and at a suitable rotation angle. Initially, the SiC/Al interface was treated as a single crystal of Al and a single crystal of SiC, with an initial gap between them. The size of the gap was determined based on the following criteria: the Al–C bond length of 1.95 Å was used for C-terminated interfaces, the Al–Si bond length of 2.41 Å was used for Si-terminated interfaces, and the average of the Al–C and Al–Si bond lengths, which was 2.18 Å, was used for nonpolar SiC interfaces [43].

The current model comprised a dual-layer nanocomposite made up of SiC and Al. The lattice constants for face-centered cubic (fcc) Al and cubic 3C-SiC were 4.0495 Å and 4.348 Å, respectively. Additionally, the lattice parameters for hexagonal 6H-SiC were *a = b =* 3.081 Å, *c =* 15.120 Å, *α = β =* 90°, and *γ* = 120°. The diffusion analysis of the system required substantial computational effort. Consequently, selecting the appropriate dimensions for the simulation box became crucial for reasonable accuracy and computational time. Different dimensions were explored to arrive at such an acceptable size, and ultimately, the dimensions depicted in Figure 2 were chosen for the simulation box. The typical dimensions of the MD model were approximately 206.4 × 86 × 278.5 Å in the *x*, *y*, and *z* directions with a total of 382,339 atoms. The chosen dimension was also adequate to ensure that the extracted cohesive zone law remained independent of the crack length during the steady-state crack propagation stage. Figure 2 illustrates a schematic representation of a C-terminated 6H-SiC/Al model, along with its dimensions and coordinate system. This configuration was used as the initial stage of the MD simulations. In order to eliminate edge effects, periodic boundary conditions were applied on the *x*- and *y*-directions, and non-periodic boundary conditions were considered in the *z*-direction.

The dimensions of the 6H-SiC/Al and 3C-SiC/Al models were similar to those shown in Figure 2. However, the accurate dimensions for each case were specifically chosen to minimize any mismatches in the *x*- and *y*-directions. Two configurations of C- and Si-terminated at the interface were considered to investigate the effect of the atom type at the interface.

This study aimed to investigate the equivalent mechanical properties of the SiC/Al interface. Two different types of interfaces were examined. In the first category, the properties of the interface were determined without subjecting the system to any annealing process. In the second category, the properties of the interface were obtained after heating the system to a temperature of 1500 K, maintaining it at this temperature for a duration of 6.0 ns, and then cooling it back to room temperature. This heat treatment induced the formation of a diffuse interface due to the diffusion process. By comparing the results from both categories, the study aimed to reveal the influence of the annealing procedure on the characteristics of the interface.

The geometric configuration was first optimized for all samples using the conjugate gradient energy minimization algorithm with a specified energy tolerance of 1 × 10^−10^ and a force tolerance of 1 × 10^−10^ eV/Å. Next, the NVT canonical ensemble at a constant temperature of 300 K was imposed on the sample for 20 ps to adjust the volume and relax the assembled interface system. Two paths were followed depending on whether the systems underwent heat treatment.

To simulate the systems without heat treatment, after energy minimization and relaxation, a crack of length 4 nm was created in the middle of the specimen, and again, another NVT canonical ensemble at a constant temperature of 300 K was imposed on the sample for 60 ps to equilibrate the system and relieve any pre-stress after removing the atoms of the crack region. Once the equilibration process was completed, the system was ready for the loading step.

However, to simulate the systems under heat treatment, the isothermal-isobaric (NPT) ensemble at zero pressure and a constant temperature of 300 K was used for 30 ps to adjust the volume and relax the assembled interface system. Further, the sample was gradually heated to 1500 K at the heating rate of 1 K/ps. Afterward, the temperature was maintained at the given temperature for 6.0 ns. Finally, the sample was cooled down to 300 K at the rate of 0.1 K/ps. Then, the structural relaxation process was performed for 20 ps under zero pressure and 300 K to remove the internal residual stresses. The NPT ensemble with zero pressure was considered for all these processes. After cooling, a crack of length 4 nm was then generated in the middle of the specimen, and another NVT canonical ensemble at a constant temperature of 300 K was imposed on the sample for 60 ps to equilibrate the system and relieve any pre-stress after removing the atoms of the crack region. The system was now ready to apply loadings. The interested reader will find more detailed explanations of the diffusion process and the effects of the annealing temperature and time on the thickness of the diffusion region and the interdiffusion coefficients in Refs. [15,16].

It is worth mentioning that the investigation carried out by Hou et al. [44] focused on analyzing the influence of cooling rate on the solidification of liquid aluminum. The results of their study revealed that faster cooling rates facilitated the development of amorphous structures, while slower cooling rates promoted the formation of fcc crystalline structures. They also established that a cooling rate of 0.1 K/ps was required to achieve the fcc structure of aluminum during solidification. Consequently, in the present study, the EAM potential proposed by Mendelev et al. [35] was used for the diffusion process, and the cooling rate of 0.1 K/ps was utilized to cool the systems.

### 3.3. Loading Conditions

After the equilibration process, the simulation box underwent loading to generate stress–strain and traction–separation curves. All simulations were performed at a strain rate of 10^8^ s^−1^. A simulation time step of 1 fs was employed to ensure accurate results.

The simulation domain was divided into two regions, as illustrated in Figure 2. In region *b*, which comprised the atoms at the top and bottom boundaries, tensile and shear loads were applied. In the case of tensile crack propagation (mode I), the system was uniformly stretched along the *z*-direction by incrementally displacing each atom to achieve a uniform normal strain. Constant velocities of *v_z_* and *-v_z_* were imposed on the atoms situated on the top and bottom boundaries (region *b*) in the *z*-direction, respectively. The imposed global strain rate was equal to

(9) $${\dot{\varepsilon }}_{z}=2v_{z}/(L_{z}-2t_{b})$$

where *L_z_* is the size of the simulation box in the *z*-direction and *t_b_* is the thickness of the boundary layer (see Figure 2). The atoms between these boundary layers initially had velocities that uniformly increased from *−v_z_* to *v_z_*. The specific velocity assigned to each atom depended on its relative position along the *z*-direction, ranging from the lowest to the highest coordinate.

On the other hand, for shear crack propagation (mode II), the simulated sample was imposed by rigidly displacing the top and bottom boundary layers (region *b*) in the *x*-direction with a velocity *v_x_* and *−v_x_*, respectively, which was constant in time. The imposed shear strain rate is given by

(10) $${\dot{\gamma }}_{xz}=2v_{x}/(L_{z}-2t_{b})$$

Atoms between the top and bottom boundary layers were assigned initial velocities in the *x*-direction proportional to their relative positions along the *z*-direction. 

The mixed-mode loading condition could be obtained by displacing the boundary layers simultaneously in the *x*- and *z*-directions. In this case, the loading angle could be defined as $\theta =\sin^{-1}(v_{x}/\sqrt{v_{x}^{2}+v_{z}^{2}})$. The loading angles 0° and 90° referred to pure tensile and pure shear loading, respectively, while other loading angles indicated inclined tensile loading.

## 4. Conclusions

The influence of the diffusion layer’s formation on the equivalent mechanical properties of the two-phase MMC interface was investigated using molecular dynamics simulations. The C- and Si-terminated 6H-SiC/Al and 3C-SiC/Al interfaces were considered in this study. A thin diffusion layer was created at the composite interface by heating it to 1500 K for 6.0 ns. The system was mechanically tested after cooling to a temperature of 300 K, which involved applying tensile, shear, and mixed-mode loadings. The traction–separation relationship of the interface, which was essential for cohesive zone modeling, was obtained. The following conclusions can be drawn from the present computations:

- The formation of the diffusion layer did not cause a significant effect on the elastic and shear moduli.
- The tensile and shear strengths of the Si-terminated interfaces were lower than those of their C-terminated counterparts before heat treatment. However, after heat treatment, the strengths of the two interfaces approached each other.
- The formation of a diffusion layer increased the tensile strength of the C- and Si-terminated interfaces by about 20% and 40%, respectively, compared with the interfaces before heat treatment.
- Following heat treatment, the work of separation increased by approximately 30% and 100% for the C- and Si-terminated interfaces, respectively.
- The shear strength was significantly lower than the tensile strength at the 6H-SiC(0001)/Al(111) and 3C-SiC(111)/Al(111) interfaces. Therefore, there was no direct correlation between shear and tensile strengths for these interfaces, unlike isotropic materials, where the shear strength was about half that of the tensile strength.
- The existing continuum-based cohesive zone model was consistent with the proposed traction–separation law based on the MD results.
- The hierarchical multiscale modeling of the interface in finite element software can be done using the cohesive zone model obtained from the MD simulations.

In general, heating the SiC/Al interface and creating a diffusion layer in a controlled environment enhances the interface’s mechanical properties, making it conducive to practical applications. It is imperative to acknowledge that any alteration in the crystal’s orientation could lead to different failure mechanisms within the nanocrystals. As a result, the findings presented in this study are particularly pertinent to the selected orientation relationships deduced from empirical observations.

## Figures and Tables

**Table 1:** The elastic modulus, maximum tensile stress, toughness, and work of complete separation of SiC/Al composites under pure tensile loading (mode I).

Composite Material	Annealing Condition	*E* (GPa)	σ_max_ (GPa)	Toughness (10^9^ J/m^3^)	Work of Separation (J/m^2^)
C-terminated 6H-SiC/Al	Before DL formation	161.7	4.39	0.174	4.81
	After DL formation	163.5	5.17	0.231	6.41
Si-terminated 6H-SiC/Al	Before DL formation	162.9	3.55	0.052	2.49
	After DL formation	164.7	5.02	0.217	5.59
C-terminated 3C-SiC/Al	Before DL formation	165.5	4.40	0.164	4.50
	After DL formation	167.4	5.40	0.183	5.71
Si-terminated 3C-SiC/Al	Before DL formation	163.7	3.57	0.071	3.17
	After DL formation	166.0	5.21	0.232	6.21

**Table 2:** The work of adhesion and critical fracture stress obtained using Griffith’s theory in Equation (2) for the C- and Si-terminated 6H-SiC/Al and 3C-SiC/Al interfaces at 300 K.

	6H-SiC/Al		3C-SiC/Al	
	C-Terminated	Si-Terminated	C-Terminated	Si-Terminated
*woa* (J/m^2^)	1.09	0.74	1.12	0.86
*σ*_max_ (GPa) (Equation (2))	5.24	4.38	5.43	4.73

**Table 3:** The shear modulus and maximum shear stress of SiC/Al composites under shear loading (mode II).

Composite Material	Annealing Condition	*G* (GPa)	τ_max_ (GPa)
C-terminated 6H-SiC/Al	Before DL formation	36.9	1.71
	After DL formation	36.5	1.80
Si-terminated 6H-SiC/Al	Before DL formation	39.2	1.12
	After DL formation	35.2	1.82
C-terminated 3C-SiC/Al	Before DL formation	39.8	1.72
	After DL formation	32.2	1.60
Si-terminated 3C-SiC/Al	Before DL formation	33.1	1.39
	After DL formation	30.7	1.65

**Table 4:** The loading angle *θ* and corresponding boundary velocities *v_x_* and *v_z_* for mixed-mode loading.

*θ* (°)	*v_x_* (Å/ps)	*v_z_* (Å/ps)
0	0	0.01225
15	0.00317	0.01183
30	0.00612	0.01060
45	0.00866	0.00866
60	0.01060	0.00612
75	0.01183	0.00317
90	0.01225	0

**Table 5:** The maximum stress, maximum normal separation, and constants *α* and *β* of the proposed CZM.

Composite Material	Annealing Condition	*σ*_max_	*δ_n_* (Å)	*α*	*β*
C-terminated 6H-SiC/Al	Before DL formation	4.39	27	0.759	0.663
	After DL formation	5.17	28	0.703	0.414
Si-terminated 6H-SiC/Al	Before DL formation	3.55	17	0.689	0.939
	After DL formation	5.02	24	0.764	0.732
C-terminated 3C-SiC/Al	Before DL formation	4.40	28	0.783	0.816
	After DL formation	5.40	28	0.932	0.859
Si-terminated 3C-SiC/Al	Before DL formation	3.57	20	0.724	0.621
	After DL formation	5.21	24	0.880	0.829

**Table 6:** The elastic constants *C*_11_, *C*_12_, and *C*_44_, bulk modulus *K*, Young’s modulus *E*, shear modulus *G*, and Poisson’s ratio *ν* determined through MD simulations using the EAM and Tersoff potential functions and compared with other MD simulations and experimental data.

Material	Method	*C*_11_ (GPa)	*C*_12_ (GPa)	*C*_44_ (GPa)	*K* (GPa)	*E* (GPa)	*G* (GPa)	*ν*
Al	Present ^a^	107.03	61.06	31.05	76.38	62.67	22.99	0.363
	Present ^b^	105.09	59.46	30.66	74.67	62.12	22.82	0.361
	MD ^c^	107.21	60.60	32.88	76.14	63.44	23.31	0.361
	Experiment ^d^	107.3	60.08	28.3	75.7	63.83	23.48	0.359
3C-SiC	Present	383.78	144.41	239.75	224.20	304.81	119.68	0.273
	MD ^e^	390.1	142.7	191.0	225.1	313.6	123.7	0.268
	Experiment ^f^	390	142	256	225	314.2	124	0.267

^a^ The EAM potential proposed by Mishin et al. [36]. ^b^ The EAM potential proposed by Mendelev et al. [35]. ^c^ Ref. [39]. ^d^ Ref. [22]. ^e^ Ref. [40]. ^f^ Ref. [21].

## Data Availability

Data available on request.
